# Efficacy of combined topical imiquimod, 5-fluorouracil, and tretinoin in the treatment of a basaloid atypical proliferation on the plantar surface in a patient who is nonsyndromic for basal cell nevus syndrome

**DOI:** 10.1016/j.jdcr.2026.02.039

**Published:** 2026-02-23

**Authors:** William J. Nahm, Evangelos V. Badiavas, Robert S. Kirsner, Claire L. Shen, George W. Elgart

**Affiliations:** a New York University Grossman School of Medicine, New York, New York; b Dr Phillip Frost Department of Dermatology & Cutaneous Surgery, University of Miami Miller School of Medicine, Miami, Florida; c Sylvester Comprehensive Cancer Center, Miami, Florida; d University of California, San Diego, California

**Keywords:** 5-fluorouracil, basal cell carcinoma, basal cell nevus syndrome, basaloid atypical proliferation, imiquimod, plantar surface, topical therapy, tretinoin

## Introduction

Palmoplantar pits are a distinctive clinical manifestation of basal cell nevus syndrome (BCNS), with histopathology revealing characteristic morphologic features and immunohistochemistry with Ber-EP4 and TDAG51 demonstrating aberrant adnexal differentiation patterns.^1^ Studies show palmoplantar pits contain basaloid cell aggregates and atypical proliferations histologically resembling basal cell carcinomas (BCCs), yet these represent a distinct clinicopathologic entity.^2^^,^^3^ The basaloid lesions of palmoplantar pits in BCNS patients rarely progress to acral BCC, though instances of BCC development have been reported, though related to secondary exposure risk factors such as radiation.^1^^,^^4^

Although basaloid atypical proliferations have been reported on the soles of BCNS patients, no clearly documented cases exist in nonsyndromic patients (not meeting BCNS criteria). Moreover, while discussions exist about treatments for BCCs on the soles, management of basaloid atypical proliferations in this location remains undescribed.^5^, ^6^, ^7^ Herein, we present a case of a patient who exhibited no clinically evident syndromic features of BCNS but had a growing basaloid atypical proliferation on the sole of his foot, which was treated with a course of topical imiquimod, 5-fluorouracil, and tretinoin.^8^

## Case report

**Fig 1:**
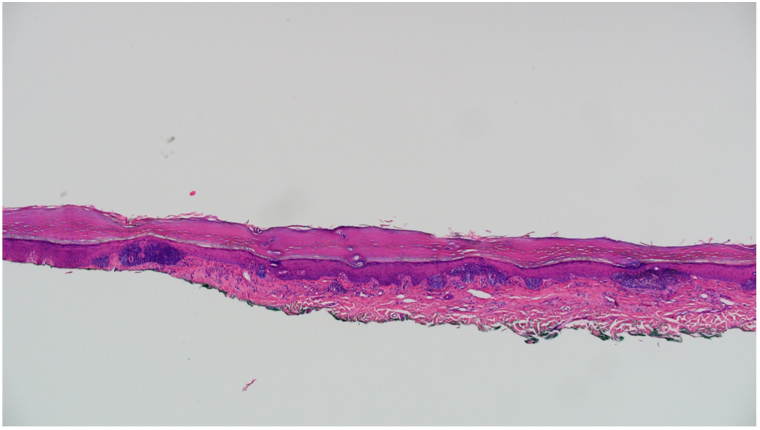
Pathology of the plantar surface of the left foot, from 5 years ago, revealing a focal epithelial atypical proliferation. Beneath the basal layer of the epidermis at several points, there is a basal epidermal proliferation of slightly more prominent atypical cells organized into irregular sheet-like collections. There is no obvious clefting beneath the tumor aggregates, and at several points overlying them, there is extensive parakeratosis. (original magnification, hematoxylin and eosin, ×40).

An 84-year-old male presented with a growing plaque on the left plantar foot for over 28 years. A shave biopsy of the lesion 5 years prior demonstrated basal epidermal proliferations of slightly more prominent atypical cells organized into irregular, sheet-like collections below the basal layer of the epidermis, with no obvious clefting beneath the tumor aggregates and prominent overlying parakeratosis at several points (Fig 1). Concerned about BCC transformation, the patient used imiquimod for 24 weeks, resulting in minimal inflammation and slight lesion reduction; he then left the lesion untreated for 5 years.

**Fig 2:**
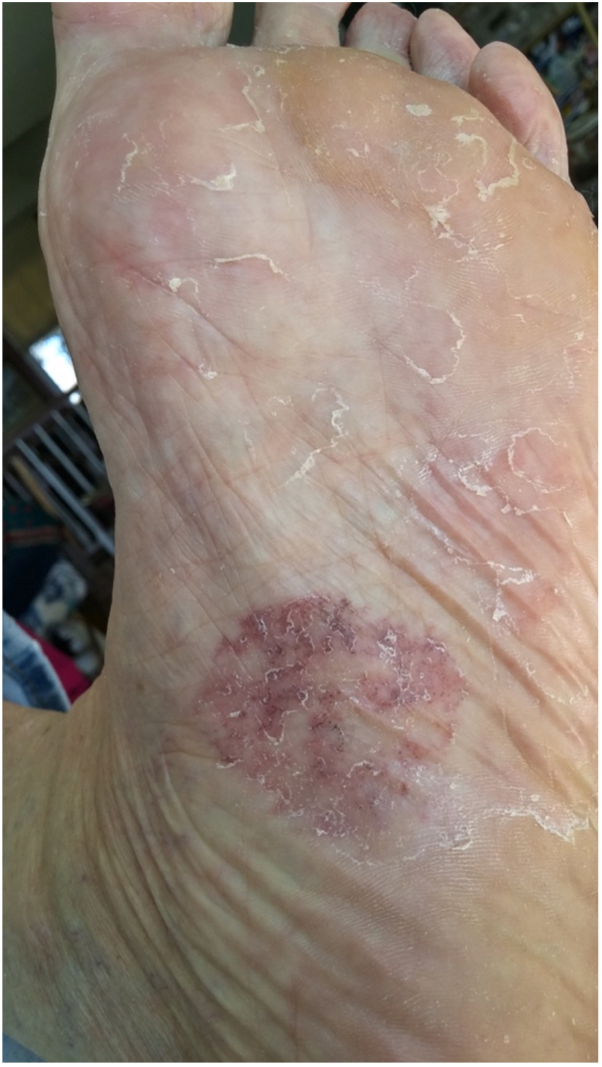
Pretreatment image of the basaloid atypical proliferation on the sole of the left foot.

**Fig 3:**
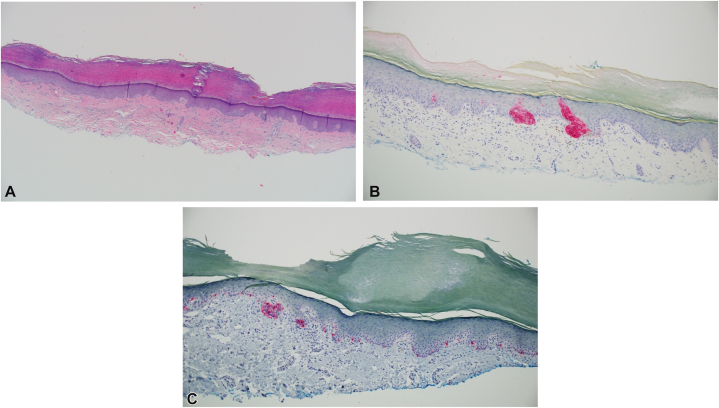
Pretreatment pathologic images of the basaloid atypical proliferation. **A,** Pathology of this lesion revealed the presence of a focal basaloid atypical proliferation with focal junctional melanocytic collections. The foci of basaloid islands, along the base of the specimen, were somewhat altered, displaying a clear appearance. **B,** Immunoperoxidase to Ber-EP4 highlights the basaloid proliferation. **C,** Melan-A immunostaining demonstrates unremarkable melanocytic proliferation. (original magnification; **A,** hematoxylin and eosin, ×40; **B,** Ber-EP4 stain ×100; **C,** Melan-A stain, ×100).

The presenting lesion was a slightly crusty, erythematous, variegated plaque measuring 3.3 cm × 3.5 cm on the medial plantar surface of the left foot (Fig 2). It had slowly grown from a pea-sized macule, becoming moderately uncomfortable with weight-bearing and occasionally having pinpoint bleeding after scratching. A shave biopsy of the lesion demonstrated a focal basaloid atypical proliferation (similar to pathology from 5 years ago) with a focal junctional melanocytic collection. The foci of basaloid islands at the base of the specimen were somewhat altered but displayed a clear, but proliferative, appearance (Fig 3, *A*). Immunoperoxidase studies showed positivity for Ber-EP4 in the basaloid aggregates (Fig 3, *B*). Immunostaining for Melan-A delineated unremarkable melanocytic proliferation.

Beyond multiple BCCs, history and examination revealed no other syndromic features of BCNS (frontal bossing, hypertelorism, odontogenic keratocysts of the jaw, skeletal abnormalities, medulloblastomas, meningiomas, or cardiac fibromas). The patient reported 12 BCCs over 35 years distributed over his head, trunk, and extremities (excluding hands and feet), with no other skin cancer types. He denied arsenic exposure or radiation treatments to the feet.

Due to discomfort caused by the lesion, the patient sought treatment but declined surgical and radiation options due to their morbidities. Although the patient had partial success with imiquimod treatments, he wanted to employ another topical regimen. A combination approach of imiquimod, 5-fluorouracil, and tretinoin was presented, and the patient used a 14-week course on the sole of his foot.^8^ The patient applied a mixture of a full packet of imiquimod 5% cream, approximately half that volume of 5-fluorouracil 5% cream, and a pea-sized amount of tretinoin 0.1% cream under a nonadherent pad and elastic wrap for 5 days a week at night for 14 weeks.

**Fig 4:**
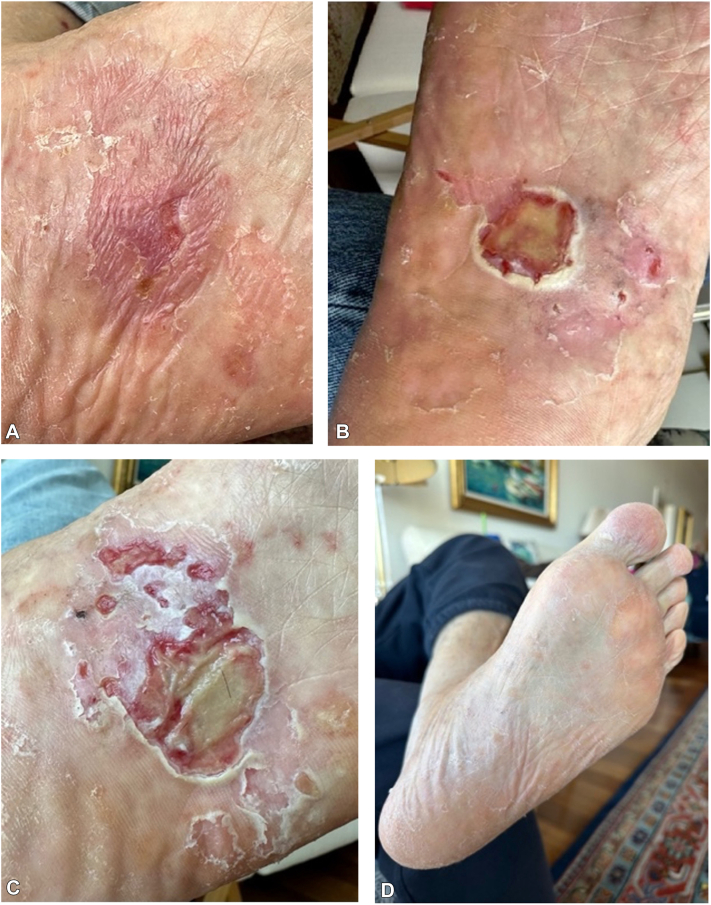
Clinical images after starting a course of imiquimod, 5-fluorouracil, and tretinoin on the sole of the left foot for 14 weeks. **A,** After 4 weeks of treatment, the treated area developed intense erythema and minimal ulceration. **B,** After 11 weeks of treatment, the area developed further ulceration with background of inflammatory scaling and erythema. **C,** After 13 weeks of treatment, the treated area developed a larger ulceration with central necrosis and multiple punctate ulcerations with background scaling. **D,** Four months after the final treatment at week 14, the sole of the foot demonstrated clinical clearance of the atypical proliferation.

After starting the application, the patient exhibited an inflammatory response and presented with minimal ulceration and erythema after 4 weeks of application (Fig 4, *A*). The treated area developed increasing ulceration and inflammatory scaling over several weeks of treatment (Fig 4, *B* and *C*). After stopping therapy at week 14, the patient demonstrated complete clinical resolution of the atypical proliferation (Fig 4, *D*). The clearance of the proliferation has been present for over 2 years, and the patient reports no sequelae and the ability to bear weight on the left foot without any discomfort.

## Discussion

Our patient demonstrated a basaloid atypical proliferation on the sole, characteristic of that seen in BCNS. These lesions and BCCs in BCNS can stain with Ber-EP4.^5^ PHLDA1 (TDAG51) staining, which is positive in follicular-derived tumors but generally negative in BCCs, was not performed; its addition could have further characterized the lesion.^9^

There is a lack of clear documentation of these palmoplantar basaloid atypical proliferations in nonsyndromic patients. Also, this long-standing plantar basaloid atypical proliferation remained stable and did not progress to a BCC, consistent with the typical behavior of those palmoplantar lesions in BCNS.^1^ This lesion, combined with a history of multiple BCCs on the trunk, extremities, and head, suggests that a comprehensive mutation spectrum could have given rise to BCCs in our patient through multiple genetic mechanisms, including those of the Hedgehog signaling pathway, which are commonly the central driver in both hereditary and sporadic cases.

Excluding BCNS based solely on clinical criteria has limitations, as mild, incomplete, or mosaic forms may not meet diagnostic criteria due to variable expressivity.^10^ Basaloid follicular hamartoma represents an additional BCNS manifestation, both sharing Hedgehog pathway mutations.^10^ Without genetic testing for PTCH1, SUFU, or PTCH2 mutations, a mild or mosaic form of BCNS cannot be excluded in this patient.

Basaloid atypical proliferations in palmoplantar pits are distinct from classic superficial BCCs. Clinically, they present as palmoplantar pit-like depressions rather than raised pearly nodules on sun-exposed areas.^2^ Histologically, they lack tumor-stromal retraction artifact and demonstrate basaloid aggregates with aberrant adnexal differentiation.^1^ While both lesion types can express Ber-EP4, palmoplantar lesions can additionally demonstrate PHLDA1/TDAG51 positivity, reflecting their unique differentiation.^9^ Molecularly, both involve Hedgehog pathway dysregulation through different mechanisms: germline PTCH1 mutations in BCNS versus predominantly somatic mutations in sporadic BCCs.^2^^,^^3^^,^^9^

The limited efficacy of imiquimod monotherapy for the basaloid atypical proliferation administered 5 years prior may be attributed to reduced drug penetration through the characteristically thick skin of the plantar area. In contrast, the triple combination therapy (imiquimod, 5-fluorouracil, and tretinoin) demonstrated superior efficacy despite requiring an extended 14-week treatment course without adjuvant cryotherapy, compared to the standard 6-week regimen with cryotherapy typically reported for keratinocyte carcinoma treatment.^8^

## Conflicts of interest

None disclosed.
